# Vitamin D receptor and epigenetics in HIV infection and drug abuse

**DOI:** 10.3389/fmicb.2015.00788

**Published:** 2015-08-19

**Authors:** Nirupama Chandel, Ashwani Malhotra, Pravin C. Singhal

**Affiliations:** Feinstein Institute for Medical Research, Hofstra North Shore LIJ School of Medicine, New York, NY, USA

**Keywords:** vitamin D receptor, vitaimin D, HIV, opioids, alcoholism, epigenetics, gene expression regulation, cocaine

## Abstract

Illicit drug abuse is highly prevalent and serves as a powerful co-factor for HIV exacerbation. Epigenetic alterations in drug abuse and HIV infection determine expression of several critical genes such as vitamin D receptor (*VDR*), which participates in proliferation, differentiation, cell death under both physiological and pathological conditions. On that account, active vitamin D, the ligand of VDR, is used as an adjuvant therapy to control infection, slow down progression of chronic kidney diseases, and cancer chemotherapy. Interestingly, vitamin D may not be able to augment VDR expression optimally in several instances where epigenetic contributes to down regulation of *VDR*; however, reversal of epigenetic corruption either by demethylating agents (DACs) or histone deacetylase (HDAC) inhibitors would be able to maximize expression of *VDR* in these instances.

## Introduction

Drug abuse is an important public health problem in the United States, affecting both the user and their families. Drug abuse and addiction not only cost the United States over six billion annually in health care but are also associated with loss of productivity due to associated factors (NIDA, 2008). Illicit drug abuse is considered the second most common cause of human immunodeficiency virus type 1 (HIV-1) infection (Pandhare, 2011). Drugs of abuse such as opioids are known to produce numerous physical abnormalities, including addiction, physical dependence, withdrawal, imbalance in the Th1 pro-inflammatory and Th2 anti-inflammatory cytokines, organ damage, and epigenetic changes (Cabral, 2006; Renthal and Nestler, 2009; Wang et al., 2011). Prevalence of drug abuse is common in individuals who have experienced a stressful life and epigenetic modifications (Cadet, 2014). Epigenetics is the heritable and reversible change in gene transcription that doesn’t change DNA sequences. There are two most commonly studied epigenetic alterations, histone acetylation and DNA/histone methylation (Robertson, 2005; Renthal and Nestler, 2009). One of the primary components of epigenetics is chromatin accessibility [open (euchromatin, uncoiled, active, or permissive) or closed (heterochromatin, coiled, inactive, or restrictive)]. Chromatin and its components are nucleosomes and histones, which are involved in DNA condensation and are critically important in gene regulatory control and their expression (Takizawa, 2008; Bergman and Cedar, 2009; Robison and Nestler, 2011). Epigenetic mechanisms reversibly modulate the structure of chromatin, thereby controlling the expression of genes such as vitamin D receptor (VDR).

## Vitamin D receptor and Epigenetics

**FIGURE 1:**
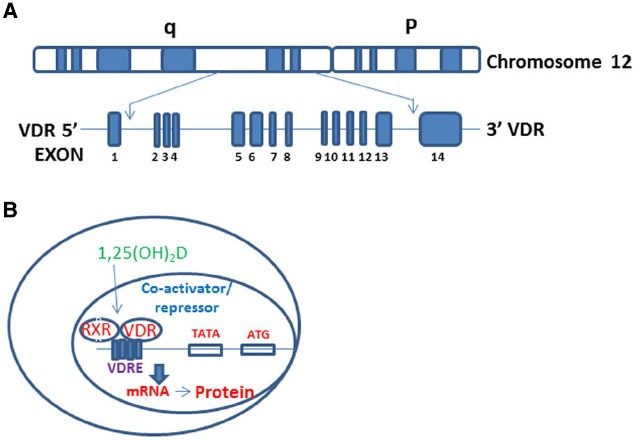
**VDR gene structure and regulation VDR target genes. (A)** The genomic structure of the *VDR* gene on chromosome 12q13. The *VDR* gene spanning the 14 exons of the *VDR* gene. **(B)** Vit D binds VDR in the cytosol facilitating its translocation into the nucleus, followed by heterodimerization with RXR. VDR-RXR complex binds to vitamin D response elements (VDREs) on target genes leading to formation of either coactivator (with liganded VDR) or repressor (with unliganded VDR) complexes.

Vitamin D (Vit D, 1, 25-α [OH]_2_D_3_) is the hormone synthesized in the skin following to sun exposure while vitamin D2 is a synthetic form and often found in fortified food. To become biologically active (1, 25-α [OH]_2_D_3_), the vitamin D undergoes a series of enzymatic conversions in the liver and kidneys (Koren, 2006). Vit D works through its receptor- *VDR*- which spans 11 exons on the reverse strand of chromosome 12q12-q14 and carries a large non-coding region containing exons 1F-1C and exon 2-9 and encoding a protein comprised of 424-amino acids (Figure 1A; Uitterlinden et al., 2004a,b). VDR is a member of the nuclear receptor (NR) family of transcription factors (Germain et al., 2006; Norman, 2007). Actions of NRs use cyclical gene regulation in which transcription factors oscillate between on and off states (Germain et al., 2006). VDR differs from these classical NRs by being located in the nucleus even in the absence of its ligand (1, 25-α [OH]_2_D_3_) (Klopot et al., 2007; Cantorna and Waddell, 2014; Singh et al., 2015); however, the presence or absence of VDR ligand determines whether it will recruit activator or repressor complexes (Tagami et al., 1998; Singh et al., 2015). VDR heterodimerizes with Retinoid X Receptor (RXR) and forms VDR-RXR complex, which recruits either repressor or activator complexes depending on its unliganded or liganded status (Figure 1B; Prufer et al., 2000; Pike et al., 2012; Singh et al., 2015). VDR ligand status modulates proteosomal degradation as well as regional chromatin profile through enzymatic control of histone modifications (Goldberg et al., 2007; Peleg and Nguyen, 2010; Kongsbak et al., 2013).

VDR expression in unstimulated T cells is controversial, whereas activated T cells show robust expression (Provvedini and Manolagas, 1989; Palmer et al., 2011; Joseph et al., 2012). VDR also modulates the immune response via balancing Th1 /IL17 axis and Th2/Tregs axis (von Essen et al., 2010; Cantorna and Waddell, 2014). Liganded VDR acts as negative regulator of renin (Li, 2003; Salhan et al., 2012), which generates angiotensin (Ang) II, a hall mark of Vit D deficient state (Chandel et al., 2013b). Ang II is a vasoactive agent and is known to induce oxidative stress in a variety of cells, including T lymphocytes (Shah et al., 2010; Chandel et al., 2012, 2013a; Rehman et al., 2013; Rai et al., 2015).

Fetahu et al. (2014) clearly elaborated the importance of methylation and acetylation on the regulation of *VDR* and other vitamin D target genes. Vit D induces VDR histone acetylation through histone acetyl transferase to enhance transcription of VDR (Karlic and Varga, 2011). In two colorectal cell lines, HCT116 and SW480, knockdown of HDAC3 not only restored the *VDR* expression but also preserved the sensitivity of *VDR* to Vit D (Godman et al., 2008). Vit D increases H3K27 acetylation on the promoter of several early target genes of VDR (Seuter et al., 2013) specially on p21 promotes in MDA-MB453 breast cancer cell line (Saramäki et al., 2009).

**FIGURE 2:**
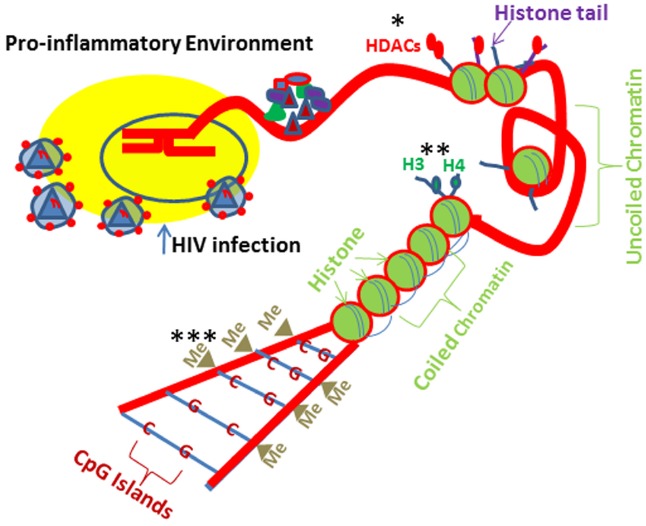
**HIV-1 and drugs of abuse induced epigenetic Changes.** Cells exposed to HIV-1 and drugs of abuse lead to similar epigenetic modifications. Drug and HIV milieus promote pro-inflammatory environment and induce epigenetic changes at the histone/DNA levels. Alcohol induces histone H3 and H4 acetylation. In contrast, morphine can lead to HDAC1 and HDAC2 recruitment to the histones preventing gene transcription. Morphine can cause epigenetic changes at the DNA level potentially resulting in increases in DMNT (DNA methyltransferase) recruitment to the DNA, leading to CpG methylation, which would prevent specific gene expression. *HDACs (histone deacetylases); **H3 and H4 (histone fractions); ***Me (methyl group).

DNA methylation, which is catalyzed by DNA methyl transferases (DNMTs), is the most studied epigenetic mechanism contributing to down regulation of gene transcription and maintains chromatin in its inactive state through Cytosine–Phosphate-Guanine (CpG) repetitive sequences (Okano et al., 1998; Herman and Baylin, 2003; Wang and Leung, 2004; Robertson, 2005; Figure 2). Vit D has been demonstrated to rescue DNA methylation in a site specific manner (Doig et al., 2013) such as VDR promoter region in HIV milieu (Chandel et al., 2013a). Age related CpG methylations of rectal mucosal genes have been shown to be influenced by Vit D status (Tapp et al., 2013). In females, plasma Vit D levels and gene-specific methylation correlated negatively. Unlike DNA methylation, which down regulates gene expression, histone methylation is able to repress as well as activate the gene transcription, depending on the site and degree of methylation (Bergman and Cedar, 2009). A regulatory effect between VDR and histone demethylases has been found in colon cancer cell line, SW-480 (Pereira et al., 2012a,b). Yamane et al. (2007) showed a positive correlation of a lysine demethylase (KDM6B) with VDR and a negative correlation of KDM6B with Snail in patients with colon cancer. Breast cancer cells displayed low VDR and enhanced methylation at *VDR* promoter; nonetheless, treatment with a DNMT inhibitor (5-Aza 2 -deoxycytidine) not only down regulated DNA methylation but also restored *VDR* mRNA expression (Marik et al., 2010). On the other hand, neither Vit D nor demethylating agent (DAC) restored VDR expression either in densely methylated VDR in choriocarcinoma trophoblastic cell lines (JEG-3 and JAR; Novakovic et al., 2009) or in colon cancer cells lacking VDR (Höbaus et al., 2013). Similarly, parathyroid tumors displayed decreased VDR expression without any alterations in methylation (Gogusev et al., 1997; Varshney et al., 2013). Interestingly, both DACs and histone deacetylase inhibitors (HDAC-IN) activated bone morphogenetic protein2 (BMP2, a key hormone responsible for maintenance of bone metabolism) in combination with Vit D (Fu et al., 2013). Lin et al. (2008) reported that methyltransferase (*EZH2*) may increase the H3K27 trimethylation at *VDR* promoter causing the suppression of target gene in colorectal cancer cells. Vit D is also demonstrated to cause attenuation of methylation on *WNT* genes (Rawson et al., 2012), *e-cadherin* (Lopes et al., 2012), and PDZ-LIM domain containing protein 2 promoter (Vanoirbeek et al., 2014).

### HIV, VDR, and Epigenetics

HIV-infected T cells have been reported to have attenuated expression of VDR through enhanced cytosine methylation at *VDR* promoter (Chandel et al., 2013a). Optimal expression of T cell VDR expression was achieved with the use of Vit D and a DAC (azacytidine) together. These investigators showed that down regulation of VDR was associated with elevated renin and Angiotensin II levels; furthermore, inhibition of VDR methylation not only restored the VDR expression but also provided the protection against HIV-induced T cell apoptosis (Chandel et al., 2013a). In these studies Vit D also restricted HIV replication in T cells. This effect of Vit D is consistent with the hypothesis forwarded by Li (2003)—Vit D- is a negative regulator of renin. Interestingly, renin and HIV protease are structurally similar (Chandel et al., 2014), therefore, renin enhances HIV replication by cleaving HIV-Gag (substrate for HIV protease; Scharpe et al., 1991; Chandel et al., 2014) and HIV protease increased Angiotensin I by cleaving angiotensinogen (Hyland and Meek, 1991). In these studies, up regulation of VDR not only inhibited HIV replication but also down regulated renin generation in T cells (Chandel et al., 2014). In the past, aging HIV infected patients used to have low blood pressure, partly because of cachexia and associated intestinal parasitic infections (Mattana et al., 1999; Hejazi et al., 2013). Currently, these patients are developing metabolic syndrome and hypertension (Pandey et al., 2008). However, it is not clear whether development of hypertension in HIV infected patients is related to HIV-induced activation of renin angiotensin system. Nonetheless, protease inhibitors will be able to control both residual viral load as well as blood pressure. Similarly, use of renin inhibitors or VDR agonists will not only control blood pressure but would also slow down HIV replication. At present, silenced HIV genomes and the latent HIV reservoirs are major obstacles for viral eradication strategies. Epigenetic mechanisms seem to play multiples roles in HIV latency (Pearson et al., 2008; Taube and Peterlin, 2013), including HIV promoter silencing, transition of activated CD4^+^ T-cells to resting memory CD4^+^ T-cells, and post-transcriptional modifications during infection (Siliciano and Greene, 2011).

Silverstein et al. (2011); Donahue et al. (2013) highlighted the role of methamphetamine (MA) toxicity in HIV infection. HIV and MA are more toxic together and both affect the same region of brain; additionally, MA crosses through blood brain barrier easily. These investigators showed that HIV gp120 is the main protein which causes neurotoxicity and cell death and drugs of abuse like morphine, cocaine and MA have the potential to interact with gp120 and multiply its effect to many folds. Later Silverstein and Kumar (2014) showed that both *in vitro* and *in vivo*, exposure to alcohol and HIV proteins results in increased levels of expression of pro-inflammatory cytokines such as interleukin-1 β and tumor necrosis factor-α, along with increased levels of oxidative stress.

### Ethanol, VDR, and Epigenetics

Alcohol abuse symptoms often have epigenetic background. The anxiolytic effects of acute alcohol ingestion occur in conjunction with the down-regulation of HDAC activity and up-regulation of H3/H4 acetylation and cAMP binding protein (CBP; Lefevre et al., 1995). HIV-infected patients are twice likely to consume alcohol as the general population (Pandrea et al., 2010). Chronic alcohol consumption has been found to increase viral replication and augmentation of pro-inflammatory cytokine production in HIV-infected patients (Rosenbloom et al., 2010). Individuals suffering from both HIV and alcoholism show far greater brain abnormalities compared to those afflicted with one condition alone (Kelkar et al., 2002). D’Addario et al. (2013) showed that binge drinking induced alterations in the expression of prodynorphin and pronociceptin genes in the rat amygdala. These authors reported increased acetylation of histone 3 at lysine 9 (H3K9Ac) but decreased abundance of H3 trimethylation at lysine 27 (H3K27me3) residues at the promoters of these two genes after 1 day alcohol administration. However, 5 days alcohol administration showed increased of H3K9Ac only at the pronociceptin promoter. Qiang et al. (2011) reported that withdrawal from chronic intermittent administration of alcohol increased H3K9Ac at the glutamate receptor, *NR2B*. They also found decreased abundance of the methyltransferases, G9a and Suv39h1 (KMT1A), and HDAC1-3 on the *NR4B* promoter region. Ethanol has also been demonstrated to induce apoptosis in T cells through VDR down regulation (Kapasi et al., 2003; Segal and Widom, 2009; Rehman et al., 2013).

### Opiates VDR and Epigenetics

#### Morphine

Among all opioids, morphine has been frequently studied with respect to its potential epigenetic effects. Various studies highlighted the effects of HDACs in morphine induced “conditioned place preference (CPP),” the tendency for addicted animals to prefer locations associated with drug administration. Since HDAC inhibition augmented morphine-induced CPP it suggests that HDAC activity contributes to the development of addiction (Robinson and Kolb, 1997). A recent study showed that morphine induced brain derived neurotrophic factor through change of HDACs levels and increasing H3k27 trimethylation at *BDNF* promoter which led to neural plasticity (Koo et al., 2015). Morphine works through mu receptor (*OPRM1*) and its expression has been modulated by MeCP2 in mice (Meaney and Ferguson-Smith, 2010; Regan et al., 2012). In these studies, DNA methyl transferase (Dnmt) 1 led to methylation and remodeling of the OPRM1 promoter in various CNS regions. Lin et al. (2008) have shown the recruitment of HDAC1/2 *OPRM1* promoter and this effect was prevented by HDAC inhibitors such as trichostatin A. Another study demonstrated that cycloheximide activated the murine *OPRM1* gene in an epigenetic fashion and recruited the Ac- H3 and methylated H3-K4 on the promoter (Kim et al., 2011). Current consensus indicates that morphine induces T cell loss (Yin et al., 2006) through VDR down regulation and Vit D and VDR agonists carry potential to restore VDR expression and preservation of T cell integrity (Singhal et al., 1999, 2001; Chandel et al., 2012).

#### Cocaine

Cocaine induces substantial changes in brain gene expressions (Freeman et al., 2001, 2008; Albertson et al., 2006). Levine et al. (2005) documented a role of cAMP response element binding (CREB) protein (CBP) in cocaine-induced acetylation of histones by acetyl transferases at the *fosB* promoter (gene responsible for addiction). Inhibition of HDACs alters behavioral responses to drugs like cocaine. Malvaez et al. (2011) also identified a critical role of CBP in behavioral alterations as a consequence of cocaine-induced histone modifications and gene expression via acetylation. He showed CBP functionality in drug-associated memories that might have contributed to drug addiction. Romieu et al. (2008) also showed role of HDAC inhibitors on cocaine induced behavioral and molecular effects including self-administration of cocaine through modulation of acetylation. HDAC inhibitors significantly enhance locomotor activity upon drug induction (Kumar et al., 2005; Renthal et al., 2007). Mice deficient in the Histone Acetyl Transferase CBP have decreased histone acetylation and display reduced sensitivity to cocaine (Levine et al., 2005). Besides histone acetylation, cocaine also modulates histone methylation (Maze et al., 2010). Chronic cocaine administration modifies the histone H3 methylation and induces long-term cocaine effects (Maze et al., 2011). Chronic cocaine use represses histone-lysine N-methyltransferase 2 (EHMT2, also known as G9a) resulting attenuated histone methylation globally (in the nucleus) and manifesting in the form of altered behavioral responses. The inability of EHMT2/G9a to regulate gene transcription following repeated cocaine ingestion contributes to abnormal synaptic plasticity (Robinson and Kolb, 1997; Maze et al., 2010). Buch et al. (2012) have found that cocaine can be considered as a multifactorial agent that accelerates HIV-1 infection as well as its progression. They have shown that cocaine further enhances the viral-induced neurotoxicity (Sulzer et al., 2005). Schmidt et al. (2013) highlighted that the epigenetic mechanisms including histone modification and DNA methylation contribute to drug-induced gene expression profile. Cocaine also increased the expression of methyl-CpG binding protein 2 (MeCP2) and produced *de novo* DNA methylation (Taniguchi et al., 2012; Kennedy et al., 2013).

#### Amphetamine

Amphetamine is being used to treat many ailments (Opler et al., 2001; Thorpy, 2001). It can pass through the blood brain barrier and raises the level of various neurotransmitters in the brain (Torres et al., 2003; Sulzer et al., 2005; Sulzer, 2011). Chronic administration of amphetamine induces alterations in the acetylation status of histone 4 (Kalda et al., 2007; Shen et al., 2008), histone methylation at H3k9 (me)2 at *C-fos* promoter (Renthal et al., 2008) and DNA methylation at multiple (total 25) genes, including *mPFC*, *OFC*, and *Nac* (Mychasiuk et al., 2013). Deng et al. (2010) showed that amphetamine induces the phosphorylation of *Mecp2*, a gene essential for learning and memory. Vit D provided protection against the serotonin-depleting effect of amphetamine in the brain of animals in the setting of repeated dosage administration. Vit D deficient rats traveled farther in locomotion test after acute dosage administration (Cass et al., 2006; Kesby et al., 2012). Vit D treated animals also showed reduction in amphetamine-induced dopamine and its metabolites when compared to control (Cass et al., 2006). MA has also been reported to induce changes in glutamate function through epigenetics (Cadet and Jayanthi, 2013). In another study, alterations in histone acetylation were accompanied by decreased expression of HDAC1 but increased expression of HDAC2 protein levels (Martin et al., 2012). Cadet and Jayanthi (2013) provides direct evidence for epigenetic regulation of transcriptional effects of chronic MA exposure on glutamate receptors, which describes the potential roles of REST, CoREST, *MeCP2*, HDAC1, and HDAC2 in mediating MA-induced down regulation of *GluA1*, *GluA2*, and *GluN1* transcription levels. They showed the MeCP2 and CoREST induced recruitment of HDAC2 onto the chromatin, resulting H4K5, K12, and K16 deacetylation and decreased H4K5ac, K12ac, and K16ac binding onto Glutamine11 and 2 DNA sequences. Histone H3 methylation at the Fobs promoter after repeated amphetamine exposure led to decreased transcription (Renthal et al., 2008). Consistent with these results, increased expression of the histone H3 methyltransferase JMJDs is also an after effect of chronic amphetamine exposure (Renthal et al., 2008).

Repeated injections of marijuana also caused decreased H3K9me2 and increased H3K4me3 at sites flanking the proenkephalin of the rat (Tomasiewicz et al., 2012).

## Conclusion and Future Direction

HIV and drugs provide an environment which is conducive to short term and long term epigenetic modifications leading to alterations in gene expression. Epigenetic alterations are also dependent on use of single or multiple drugs. Since both HIV and drugs such as morphine modulate the immune system, environment in these scenarios is likely to be complicated by ongoing opportunistic infections including, bacterial, viral, and fungal. These infections may themselves carry potential to modulate epigenetics. Therefore, epigenetics is a complex issue in drugs of abuse in general and specifically in the presence of HIV infection. However, epigenetic alterations are reversible and thus strategies can be developed to reverse them.

### Conflict of Interest Statement

The authors declare that the research was conducted in the absence of any commercial or financial relationships that could be construed as a potential conflict of interest.
